# An Electrochemical Fiveplex Biochip Assay Based on Anti-Idiotypic Antibodies for Fast On-Site Detection of Bioterrorism Relevant Low Molecular Weight Toxins

**DOI:** 10.3390/toxins11120696

**Published:** 2019-11-28

**Authors:** Katharina Schulz, Christopher Pöhlmann, Richard Dietrich, Erwin Märtlbauer, Thomas Elßner

**Affiliations:** 1 Bruker Daltonik GmbH, Permoserstr. 15, 04318 Leipzig, Germany; Katharina.Schulz@bruker.com (K.S.); C.Poehlmann@bruker.com (C.P.); thomas.elssner@bruker.com (T.E.); 2 Department of Veterinary Sciences, Faculty of Veterinary Medicine, Ludwig-Maximilians-Universität München, Schönleutnerstr. 8, 85764 Oberschleißheim, Germany; r.dietrich@mh.vetmed.uni-muenchen.de

**Keywords:** electrochemical biochip, multiplex on-site detection, anti-idiotypic antibodies as epitope-mimicking reagents, bioterrorism, low molecular weight toxins, saxitoxin, microcystins, trichothecenes, aflatoxins

## Abstract

Modern threats of bioterrorism force the need for multiple detection of biothreat agents to determine the presence or absence of such agents in suspicious samples. Here, we present a rapid electrochemical fiveplex biochip screening assay for detection of the bioterrorism relevant low molecular weight toxins saxitoxin, microcystin-LR, T-2 toxin, roridin A and aflatoxin B1 relying on anti-idiotypic antibodies as epitope-mimicking reagents. The proposed method avoids the use of potentially harmful toxin-protein conjugates usually mandatory for competitive immunoassays. The biochip is processed and analyzed on the automated and portable detection platform pBDi within 13.4 min. The fiveplex biochip assay revealed toxin group specificity to multiple congeners. Limits of detection were 1.2 ng/mL, 1.5 ng/mL, 0.4 ng/mL, 0.5 ng/mL and 0.6 ng/mL for saxitoxin, microcystin-LR, T-2 toxin, roridin A or aflatoxin B1, respectively. The robustness of the fiveplex biochip for real samples was demonstrated by detecting saxitoxin, microcystin-LR, HT-2 toxin, roridin A and aflatoxin B1 in contaminated human blood serum without elaborate sample preparation. Recovery rates were between 52–115% covering a wide concentration range. Thus, the developed robust fiveplex biochip assay can be used on-site to quickly detect one or multiple low molecular weight toxins in a single run.

## 1. Introduction

Human intoxications caused by low molecular weight toxins, such as cyanotoxins and mycotoxins, are global phenomena occurring mostly through contact with contaminated food or from environmental exposure. Among the cyanotoxins, paralytic shellfish poisoning (PSP) toxins and microcystins (MCs) are two toxin groups encompassing the highest number of toxic structural variants. PSP toxins are chemically related neurotoxins comprising up to 58 congeners with saxitoxin (STX) being the most toxic one [1]. Most of the PSP poisoning events have been reported from the consumption of marine-derived seafood which can even contain lethal concentrations of PSP toxins [2], whereas intoxications caused by MCs are a result of contaminated drinking water [3]. MCs are cyclic heptapeptides characterized by their potent hepatotoxic properties [4]. Especially, the high variation of the L-amino acid residue at position two and four of MCs leads to up to 246 congeners, with the highly toxic microcystin-LR (MC-LR) being the most common congener [5]. Another health threat is associated with intoxication events caused by two groups of mycotoxins, the cytotoxic trichothecenes and carcinogenic aflatoxins. While T-2 toxin (T-2) and HT-2 toxin (HT-2) belong to the type A trichothecenes constituting the majority of contaminants in food, the macrocyclic type D trichothecenes including roridin A (RoA) have attracted more attention as an indoor pollutant [6]. Among the 20 identified aflatoxins, aflatoxin B1 (AFB1) is the most toxic and most commonly occurring toxin of this group associated with food contamination [7]. Due to a high toxicity, stability and accessibility, STX, MCs, T-2 and aflatoxins are regarded as potential biological warfare agents [8], and therefore, noted in the list of human and animal pathogens and toxins for export control of the Australia Group [9]. Additionally, STX is listed in Schedule 1 of the Chemical Weapons Convention. Exposure to these low molecular weight toxins leads to rapid onset (min to h) of non-specific symptoms ranging from headache, nausea, vomiting and diarrhea to muscle paralysis and coma in severe cases [10]. In case of a deliberate release of such agents, rapid screening tools must access the exposure level of individuals to prominent biological agents. Thus, testing biological fluids like blood serum for the presence of multiple toxins is preferable to assess a poisoning event. Multiple case reports documented the presence of the cyanotoxins STX and MCs in serum samples covering concentrations of 4.9–14 ng/mL during acute illness for STX [11] or up to 130 ng/mL MC-LR in fatal cases [12]. Serum samples from healthy individuals exposed to environmental molds were tested positive for various mycotoxins, including type D trichothecenes (up to 82 ng/mL) [13] and AFB1 (up to 0.492 ng/mL) [14]. HT-2, the primary metabolite of T-2, was detected in blood of animals exposed to T-2 [15].

Detection of multiple low molecular weight toxins is often achieved by multi-class methods utilizing chromatographic and/or mass spectrometry-based approaches [16]. While these methods achieve exceptional detection sensitivities, they all suffer from the common requirement of expensive laboratory equipment, skilled operators and complicated multi-step sample pretreatments to perform analysis. To provide a sensitive and easy-to-use detection system with fast response times, a variety of screening approaches based on immunoassay methods combined with different signal transduction principles have been developed, including magnetic microsphere-based assays [17] or fluorescence polarization assays [18]. While offering a high degree of multiplexing, they mostly rely on complex optics or moving parts and are, therefore, also not ideally suited for use in the field [19]. For point-of-care or on-site testing for medical diagnostics or food and environmental monitoring, lateral flow immunoassays (LFAs) are the best-known low cost, rapid and easy-to-use testing systems, but mostly suffering from insufficient analytical sensitivity [20]. As a consequence, intensive research was focused on alternative electrochemical transduction of biological binding events, which offer high sensitivity along with cost- and time-effectiveness [21]. Another advantage of electrochemical biochips is the potential for miniaturization and portability as well as their simple integration in low-cost devices [22]. One major analytical challenge of on-site immuno-based detection of low molecular weight toxins is the generation of broad-specific antibodies recognizing a variety of congeners within a toxin group. However, the used antibodies must also be highly specific and should not detect any unrelated toxins [23]. Therefore, most desirable for on-site testing would be a broad-reactive immuno-based assay capable to detect all congeners of the respective toxin group.

All these immunoassay-based approaches rely on a competitive test format, because haptens do not have multiple epitopes for the simultaneous binding of two antibodies. Therefore, conjugation of the toxin to carrier proteins used for coating or to a label competing with the free toxin in the sample for binding to toxin specific capture antibodies is required, which can be considered as a major drawback of competitive assays applied to detection of low molecular weight toxins. Synthesis of toxin-protein conjugates can be time-consuming due to complicated coupling procedures leading to randomly cross-linked and unstable molecules which can reduce assay sensitivity [24]. Especially for MCs and aflatoxins, manipulation of these carcinogenic compounds could be hazardous for operators and the environment. Due to coverage of STX by the Chemical Weapons Classification, handling and distribution of STX is also strongly regulated. Therefore, replacement of these toxic reagents by alternative recognition elements, such as aptamers [25], molecularly imprinted polymers (MIPs) [26] and intact cells [27], or by using epitope-mimicking molecules, such as synthetic peptides [28], phage-displayed peptides [29] and anti-idiotype antibodies [30], promise a more straightforward assay development. Particularly, anti-idiotypic antibodies of β-type (Ab2) raised against the paratope of the toxin specific antibody (Ab1) mimic the epitope of the target toxin and compete with the analyte for specific binding to the corresponding Ab1 [31], and can be used either as capture antigen [32], as competing detection reagent [33] or as surrogate toxin [34] in immunoassays. Previously, we reported the utilization of monoclonal Ab2 (mAb2) to develop microarrays for singleplex detection of STX, T-2/HT-2 or aflatoxin M1 (AFM1) and proved their applicability for analyzing human urine samples [35].

In this work, we present for the first time a fiveplex electrochemical biochip assay for on-site detection of low molecular weight toxins relying solely on anti-idiotypic antibodies as epitope-mimicking molecules. Moreover, due to the generation of reporter enzyme-labeled monoclonal detection antibodies used as tracer molecules, we demonstrate a straightforward assay allowing simultaneous detection of bioterrorism relevant STX, MC-LR, T-2, RoA and AFB1 as well as corresponding congeners with minimal hands-on time using the automated on-site biochip processing platform pBDi (portable BioDetector integrated) [36]. Furthermore, we demonstrated the applicability of the robust multiplex detection system to screen human serum—beside urine another relevant body fluid—for the presence of STX, MC-LR, HT-2, RoA and AFB1 after intentional or natural occurring poisoning incidents.

## 2. Results

### 2.1. Fiveplex Biochip Assay Design

**Figure 1:**
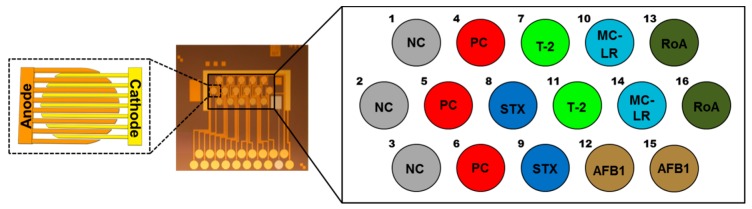
Electrical biochip based on interdigitated array (IDA) gold electrodes with fiveplex biochip spotting layout. For simultaneous detection of saxitoxin (STX; dark blue), microcystin-LR (MC-LR; light blue), T-2 toxin (T-2; light green), roridin A (RoA; dark green) and aflatoxin B1 (AFB1; olive), target specific capture mAbs were immobilized in duplicates. Abs for PC (positive control; red) and NC (negative control; grey) were spotted in triplicates. The numbers indicate the corresponding electrode position on the biochip.

In-house generated mAbs1 and mAbs2 were tested as capture and detection reagents on the electrical biochip. The ideal capture mAb (mAb1 or mAb2) as well as the concentration used for immobilization were selected during competitive tests prior to the multiplex set-up (data not shown). The utilized mAb pairs for STX, T-2 and AFB1 were the same as those characterized previously in singleplex electrochemical biochip assays [35]. Selection of the respective Ab type as capture is the crucial step regarding assay sensitivity, because the competitive reaction is dependent on the amount and functionality of the mAb on the electrode surface [35] (see also Figure S1). Suitable capture mAbs (STX/mAb2, MC-LR/mAb2, T-2/mAb1, RoA/mAb2, AFB1/mAb1) were immobilized onto the biochip in duplicates on interdigitated array (IDA) gold electrodes consisting of two interlocking sets of anodic and cathodic fingers (Figure 1). Spotting concentrations of target specific capture mAbs are shown in Table S1.

Since all specific capture Abs used are mouse monoclonal immunoglobulins (IgGs), IgGs from non-immunized mice (mouse IgG) were chosen as independent negative control (NC) in a comparable concentration. Biotinylated anti-mouse IgG (anti-mouse IgG-bio) from rabbit was spotted as positive control (PC). Control Abs were immobilized in triplicates.

To avoid the use of any toxin-protein conjugates as tracer molecules, we employed monoclonal detection antibody-β-D-galactosidase (mAb-bGAL) conjugates for the fiveplex competitive biochip assay. Applied dilutions of mAb-bGAL tracers were optimized by titration (data not shown) and optimal dilutions of mAb-bGAL tracers used in the detection mAb cocktail are presented in Table S1. Selection of the optimal tracer dilution was based on a stable maximum signal response (normalized signal close to 100%) with a coefficient of variation (%CV) less than 15%.

**Figure 2:**
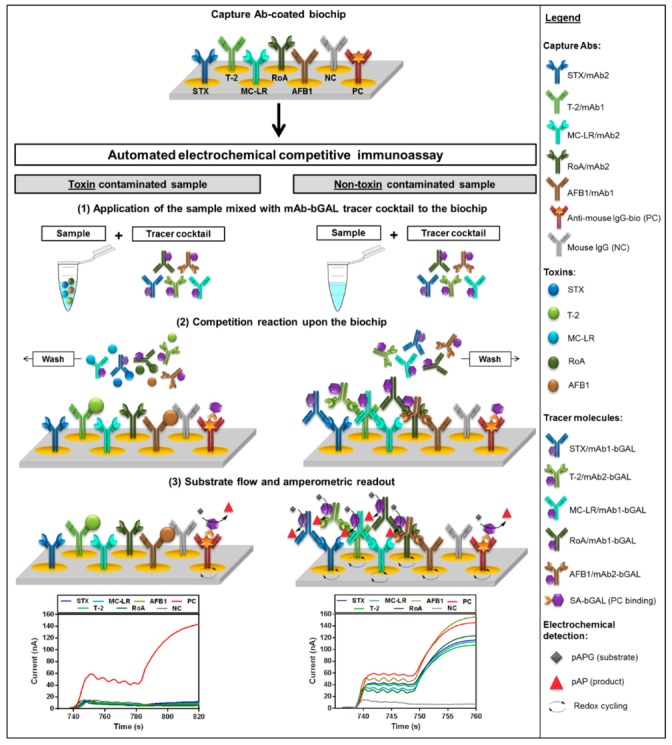
Schematic procedure of the automated fiveplex anti-idiotypic antibody-based competitive immunoassay for detection of STX, MC-LR, T-2, RoA and AFB1 upon an electrochemical biochip. (**1**) Application of the sample mixed with monoclonal detection antibody-β-D-galactosidase (mAb-bGAL) tracer cocktail to the biochip pre-coated with capture mAbs; (**2**) Competition reaction upon the biochip; (**3**) bGAL substrate flow as well as amperometric readout of each electrode position. (Note: Illustrated biochip scheme represents only a small section of the entire electrode positions).

A scheme of the assay principle for the established fiveplex electrochemical biochip applying anti-idiotypic antibodies as epitope-mimicking molecules in a competitive format is shown in Figure 2.

All pumping and incubation steps as well as electrochemical detection are performed by the fully automated on-site biochip processing platform [36]. Prior to the assay, mAb1 (T-2, AFB1) or mAb2 (STX, MC-LR, RoA) were immobilized on specific positions by physisorption on IDA gold electrodes of the biochip. Following the automated application of the sample mixed with an mAb-bGAL tracer cocktail, a competition reaction takes place upon the biochip. In case of a toxin contaminated sample, mode of competition depends on Ab type immobilized on the electrode. For detection of STX, MC-LR and RoA, free toxins in the sample compete with the immobilized mAb2 for binding to the mAb1-bGAL conjugates. In case of T-2 and AFB1, a competition between toxins in the sample and mAb2-bGAL for limiting binding sites of the immobilized mAb1 occurs. For both competition modes, the higher the toxin concentration in the sample, the fewer detection mAb-bGAL conjugates can bind to immobilized capture mAb. For a non-toxin contaminated sample, mAb-bGAL tracers are captured by the respective capture mAb. Following a washing step removing unbound molecules, detection is realized through β-D-galactosidase (bGAL) catalyzed conversion of the electrochemically inactive substrate *p*-aminophenyl-β-D-galactopyranoside (pAPG) to the electrochemically active product *p*-aminophenol (pAP), which in turn, undergoes redox cycling amplifying the signal [37]. At the same time, amperometric response curves are recorded for each electrode position. The generated current is inversely proportional to the toxin concentration in the sample due to the competitive assay principle. Regardless of whether toxins were present in the sample, PC electrode positions always show a high current signal.

In contrast to the former reported assay set-ups [35] in which biotinylated detection mAbs and a subsequent labeling with streptavidin-bGAL (SA-bGAL) were applied, here, we used covalently linked mAb-bGAL conjugates as tracer molecules. This modification resulted in a significant reduction of the assay time from 16.7 min to 13.4 min, because the additional incubation step with SA-bGAL (3.3 min) could be omitted. The automated assay program executed by the instrument is summarized in Table S2.

### 2.2. Analytical Characteristics of the Fiveplex Biochip Assay

#### 2.2.1. Assay Specificity

**Figure 3:**
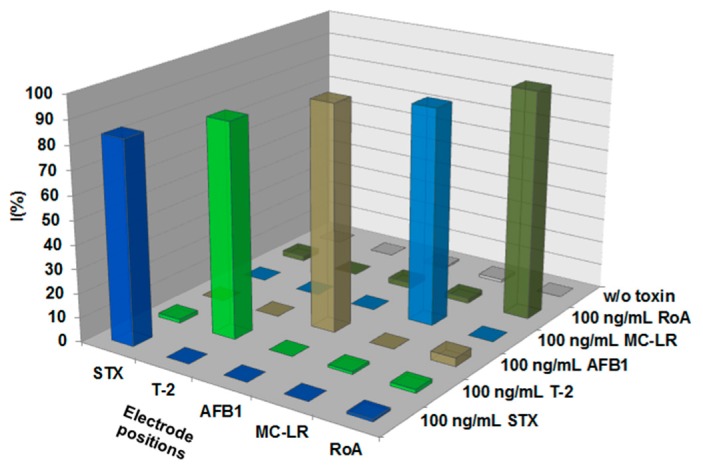
Specificity of the multiplex biochip assay for detection of STX, MC-LR, T-2, RoA and AFB1. One hundred ng/mL of each toxin was applied separately on the multiplex biochip in presence of mAb-bGAL tracer cocktail. Mean normalized signals of target electrode positions are shown as percent inhibition (%I) obtained from four independent measurements (*n* = 8, i.e., four biochips with each two target electrode positions).

A technique that can detect different types of small molecules at the same time represents a more straightforward and rapid analytical approach compared to methods detecting only one type of an analyte. However, specificity is an important parameter for the establishment of multiplex detection methods. To demonstrate the specificity of the established multiplex biochip assay for corresponding toxins, samples containing one particular toxin at a high concentration (100 ng/mL) were tested with the mAb-bGAL tracer cocktail upon the fiveplex biochip exhibiting immobilized capture mAbs on specific electrode positions. As depicted in Figure 3, only electrode positions spotted with mAb specific for the corresponding toxin revealed a high signal reduction (%I > 80%), whereas percent inhibition observed for toxins on electrode positions immobilized with non-corresponding mAbs was less than 4%. Corresponding raw data and extracted slope values are stated in Figure S2. Consequently, all tested low molecular weight toxins (STX, T-2, AFB1, MC-LR and RoA) were specifically detected as well as reliably distinguished by the established multiplex assay.

Furthermore, a method for on-site detection should ideally cover a wide range of multiple variants of one toxin group, i.e., using a broad-specific mAb to detect as much congeners as possible of PSP toxins is preferred. To assess the broad specificity of the fiveplex biochip assay, different PSP toxins, MCs or the structurally related and most abundant congener of the nodularins nodularin-R, type A trichothecenes, type D trichothecenes and aflatoxins were assayed separately at a high concentration (50 ng/mL or 100 ng/mL) in presence of mAb-bGAL tracer cocktail. Results are presented in Table 1.

MAb pairs for STX, MC-LR, RoA and AFB1 exhibit a broad specificity toward the respective toxin group, whereas the mAb pair for T-2 is highly specific for T-2 and HT-2. Data obtained for PSP toxins, type A trichothecenes and aflatoxins confirm previously published results for singleplex assays only [35]. In brief, non-hydroxylated PSP toxins (STX, dc-STX, GTX-2/-3, dc-GTX-2/-3 and GTX-5) were detected, whereas hydroxylated variants like NEO, dc-NEO, and GTX-1/-4 showed no signal reduction at the applied concentration. The mAb pair for T-2 is specific for the most prominent congeners of type A trichothecenes (T-2 and HT-2). Employed antibodies for aflatoxins recognize all five aflatoxins congeners (AFB1, AFM1, AFG1, AFB2 and AFG2) commonly found in food. For the detection of MCs, the most common and/or toxic congeners were chosen [4]. The employed mAb1/mAb2-pair specific for MC-LR recognizes at least all of the seven tested MCs (MC-LR, [DAsp^3^]MC-LR, MC-RR, MC-YR, MC-LA, MC-LY and MC-LW) as well as the structurally related nodularin-R with differing sensitivities. The variants MC-LR, [DAsp^3^]MC-LR, MC-RR, MC-YR and nodularin-R showed similar levels of signal reduction, whereas the magnitude of signal reduction was clearly lower for MC-LA, MC-LY, MC-LW indicating that arginine at position four has a great influence on antibody affinity. The mAb pair employed for the detection of type D trichothecenes (e.g., roridins, satratoxins and verrucarins) also shows a wide coverage across macrocyclic trichothecenes with distinctive specificity for roridins (RoA and RoE), followed by verrucarin A (VerA). Signal reduction in the presence of satratoxin H (SatH) was lower than for other tested type D trichothecenes suggesting that the presence of the six-membered ring within the macrocyclic ring system resulted in decreased antibody affinity [38,39].

Of particular importance is the assay’s ability to distinguish clearly type A trichothecenes and type D trichothecenes. None of the assayed type A trichothecenes were bound by the mAb pair specific for RoA, and vice versa. Thus, both mAb1/mAb2-pairs can be regarded as highly specific to their corresponding toxin group. Overall, no cross-reactivities between different toxin groups are existent, i.e., the magnitude of signal reduction for mAb pairs and non-corresponding toxins was negligible (%I < 10%).

#### 2.2.2. Assay Performance

**Figure 4:**
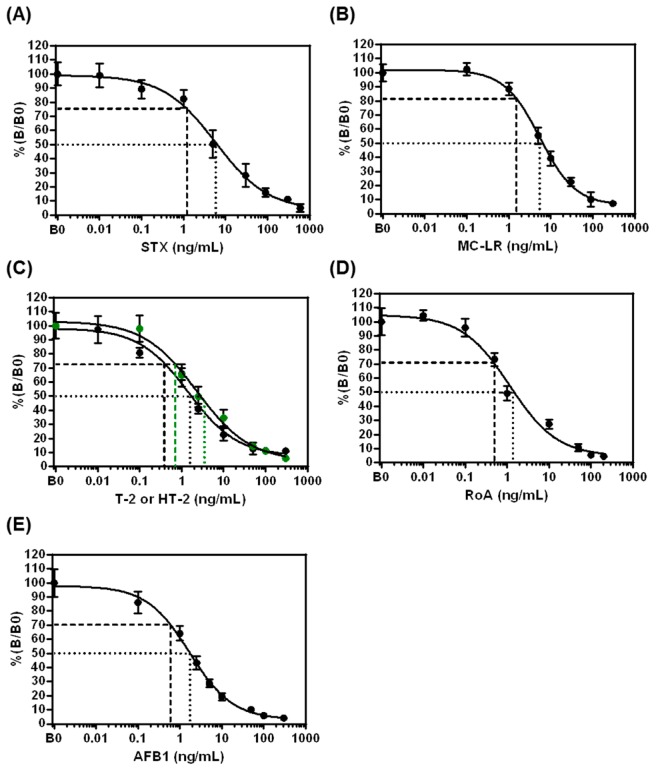
Individual dose-response curves for STX, MC-LR, T-2, HT-2 and RoA as well as AFB1 obtained with the electrochemical fiveplex biochip assay. Measurements of toxin solutions with different concentrations of (**A**) STX, (**B**) MC-LR, (**C**) T-2 (black circles) and HT-2 (green circles), (**D**) RoA and (**E**) AFB1, respectively, were performed in assay buffer (*n* = 6, i.e., three biochips with each two target electrode positions; error bars: SD). For determination of B0-values, ten biochips with each two target electrode positions were used (*n* = 20). LOD is stated as dashed line. IC_50_ is depicted as dotted line. SD = standard deviation; LOD = limit of detection.

Dose-response curves for STX, MC-LR, T-2, HT-2 and RoA, as well as AFB1 were generated by analyzing different concentrations of toxin solutions in assay buffer (Figure 4).

For all applied toxins, typical sigmoidal shaped curves with negative slopes were obtained proving that the signal is inversely proportional to the toxin concentration. Parameters describing assay sensitivity (LOD, IC_50_, IC_30_–IC_80_) obtained from dose-response curves are summarized in Table 2 indicating that the detection system is capable to detect all toxins in the low ng/mL- to pg/mL-range within a total assay time of 13.4 min. For all toxins, the working range (IC_30_–IC_80_) of the assay covered one to two orders of magnitude above the LOD.

Ten B0 and ten B_IC50_ were measured on different chips to determine the assay precision. Variability of normalized signals for individual toxin concentrations is expressed as inter-chip %CV (Table 2). In the absence of toxin (B0), inter-chip variability ranges from 9.2–11.2%. For samples with a toxin concentration at individual IC_50_ (B_IC50_), the inter-chip %CV is between 8.2 and 11.6%; thus, reproducibility of the established fiveplex biochip is demonstrated.

### 2.3. Fiveplex Detection of STX, MC-LR, T-2/HT-2, RoA and AFB1 in Buffer and Human Blood Serum

#### 2.3.1. Simultaneous Detection of STX, MC-LR, T-2, RoA and AFB1 in Buffer

**Figure 5:**
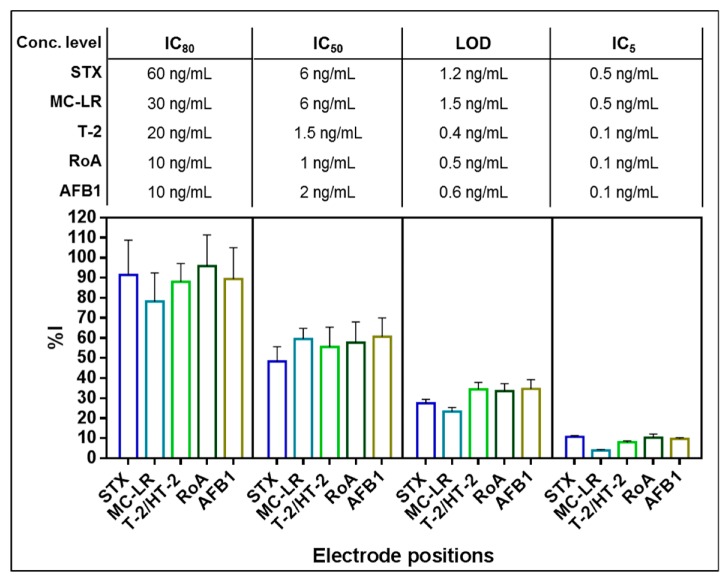
Simultaneous detection of STX, MC-LR, T-2, RoA and AFB1 in buffer. Measurements were performed with toxin mixtures containing STX, MC-LR, T-2, RoA and AFB1 close to their individual IC_80_, IC_50_, LOD and IC_5_ (*n* = 4, i.e., two biochips with each two target electrode positions; error bars: SD). Mean normalized signal of target electrode positions is depicted as %I.

In order to characterize the ability of the developed multiplex biochip assay to detect STX, MC-LR, T-2, RoA and AFB1 simultaneously, all five toxins were applied in mixtures at various concentration levels corresponding to their individual IC_80_, IC_50_, LOD and IC_5_ (Figure 5).

As depicted in Figure 5, all of the five different low molecular weight toxins were detected simultaneously using the fiveplex electrochemical biochip assay. The higher the toxin concentration used, the higher the signal reduction on each target electrode position. Signal responses in the parallel assay coincide with signals observed in biochip experiments performed with single toxin standard solutions demonstrating no significant cross-talk between different electrode positions at the tested toxin concentrations. Furthermore, it confirms the high specificity of the capture mAbs implemented on the fiveplex biochip for their respective toxin groups as shown in Section 2.2.1. Moreover, unspecific binding of different mAb-bGAL conjugates to NC immobilized with polyclonal mouse IgG was not observed (data not shown).

#### 2.3.2. Simultaneous Detection of STX, MC-LR, HT-2, RoA and AFB1 in Serum Samples

To demonstrate the applicability of the proposed fiveplex biochip assay as a rapid on-site screening tool for assessing an intentional or naturally occurring intoxication event caused by highly toxic STX, MC-LR, T-2, RoA and AFB1, parent toxins or metabolites were determined in human serum samples. Human blood serum was spiked in quintuplicate with mixtures of cyanotoxins (STX and MC-LR) and mycotoxins (HT-2, RoA and AFB1) at three different concentration levels (low: 6 ng/mL cyanotoxins, 4 ng/mL mycotoxins; mid: 20 ng/mL cyanotoxins, 10 ng/mL mycotoxins; high: 200 ng/mL cyanotoxins, 100 ng/mL mycotoxins). Additionally, five non-spiked serum samples were tested to estimate the rate of false positive results. In case of the detection of a low molecular toxin in a fortified serum, this sample was classified as correctly identified according to the assumption: %(B/B0)_Serum_ < %(B/B0)_Buffer_ at LOD. In case of blank samples, the criterion for a correct identification (i.e., as negative) is %(B/B0)_Serum_ ≥ %(B/B0)_Buffer_ at LOD. Recovery rates of spiked serum samples were determined from dose-response curves obtained in assay buffer. Results of the recovery experiments are shown in Table 3.

Despite of the complexity of the blood serum matrix, a dilution of sample matrix with assay buffer was sufficient as sample preparation for electrochemical biochip measurement. All non-spiked serum samples could be confirmed as negative (correctly identified = 100%, i.e., biochip signals for each target analyte were below LOD). Thus, no false positive results were generated. Furthermore, the established multiplex biosensor was able to detect correctly five out of five samples contaminated with low, medium and high concentration of STX, HT-2, RoA and AFB1 (correctly identified = 100%, i.e., biochip signals for stated target analytes were above LOD). For MC-LR, four out of five samples containing the low concentration (6 ng/mL) were correctly identified (80%), whereas for the medium and high concentration five out of five samples were correctly assessed as positive (100%). The final assayed concentration of MC-LR in the low concentrated serum sample is 3 ng/mL, and thus, only two-fold above LOD obtained in assay buffer (1.5 ng/mL). In combination with a recovery rate lower than 80%, this resulted probably in the false negative detection of MC-LR in one out of five samples. Recovery rates for serum samples contaminated with STX, MC-LR, RoA and AFB1 were close to 100% (STX: 103.2–113.7%, MC-LR: 78.8–98.2%, RoA: 87.9–95.6%, AFB1: 101.1–114.8%), demonstrating the high robustness of the electrochemical biochip assay. For HT-2, recovery rates are clearly lower than for other tested low molecular weight toxins ranging from 51.8–79.2% which indicate a significant matrix effect. One possible explanation might be the presence of serum compounds binding HT-2 in an unspecific manner masking the epitope for binding to the immobilized capture mAb1. This leads to an apparently increased B-value for the applied HT-2 concentration and, thus, to a decreased %(B/B0)-value in serum causing a reduced recovery rate. Nevertheless, non-spiked serum samples and serum samples spiked at the lowest HT-2 concentration of 4 ng/mL (2 ng/mL assay concentration, i.e., three-fold above LOD) could be clearly distinguished and correctly identified. 

Overall, these results indicate that the developed fiveplex biochip assay could be successfully applied for multi-toxin screening of human serum samples at relevant clinical concentrations even in the low ng/mL-range applying an automated assay procedure in a portable device.

## 3. Discussion

Low molecular weight toxins such as STX, MC-LR, T-2, RoA and AFB1 possess serious health threat to both humans and animals. Additionally, being ubiquitous in nature, they are relevant in the health and food sector as well as in the security sector. On the one hand, low molecular weight toxins are linked with natural intoxications worldwide causing severe toxicoses. On the other hand, the mentioned toxins are regarded as potential biological warfare agent and, therefore, noted in the list of human and animal pathogens and toxins for export control of the Australia Group [9]. Hence, both cases demand sensitive detection systems that are capable to detect on-site the presence or absence of these toxins in suspicious samples. Here, we present a multiplex assay suitable for fast screening of human serum samples after an acute intoxication with STX, MC-LR, T-2, RoA and AFB1. All five toxins were reliably detected in spiked serum samples over a wide concentration range from 4–200 ng/mL. However, sparse reports of human poisoning and clinical cases have been described in the literature thus far. At least a few reports of severe or fatal intoxications with STX and MC-LR, respectively, identified concentration levels of 15 ng/mL STX [11,40] and up to 130 ng/mL MC-LR [12] in serum. A case study about an acute aflatoxicosis outbreak in Africa reported high serum AFB1 levels (32.8 ng/mg aflatoxin-albumin adducts) [41]. No clinical case reports were published describing an exposition to trichothecenes with fatal outcome.

Most of the immunochemical methods for low molecular weight toxin detection employ a competitive assay format associated with a major drawback—the use of potentially harmful toxin-protein conjugates as assay reagents. Replacements of these conjugates by epitope-mimicking molecules pave a way for novel assay strategies that embodies a user-safe option to toxin-protein conjugates. However, assays based on epitope-mimicking reagents were exclusively established for single toxin detection so far. Here, we demonstrate for the first time an electrochemical multiplex biochip assay solely based on anti-idiotypic antibodies as epitope-mimicking molecules for simultaneous detection of five low molecular weight toxins (STX, MC-LR, T-2, RoA and AFB1). The newly developed fiveplex biochip assay based on a competitive immunoassay incorporates five key merits:

1. A high level multiplexing biochip to detect multiple potential bioterrorism relevant toxins in a single run.
2. An on-site applicability combined with a short assay time (13.4 min).
3. A multi-congener detection system by employing toxin group specific mAbs.
4. A completely user-safe assay due to waiving of harmful toxin-protein conjugates.
5. A straightforward assay workflow through reporter enzyme-conjugated detection mAbs.

Compared to previously reported assays for the detection of low molecular weight toxins using epitope-mimicking molecules, analytical characteristics of the here developed assay are superior (Table S3). In a previously reported assay for fumonisin B1 (FB1) [32], an anti-idiotypic nanobody (Ab2-Nb) was applied as coating antigen leading to the development of a competitive Ab2-Nb-based assay for FB1 with a 20 times higher sensitivity than that of the traditional assay with FB1-BSA conjugate. Considering the predominant traits of Nbs (small size, high stability), they fulfill on-site testing requirements of reagents for competitive immuno-based screening assays and are also promising alternative reagents to anti-idiotypic antibodies. The use of epitope-mimicking peptides, or mimotopes, is another alternative approach to substitute toxin-protein conjugates and can be obtained by phage-display and chemical synthesis. Peltomaa et al. identified a mimotope for FB1 [28] and applied it to various magnetic bead-based assays either as phage-mimotope, as synthetic mimotope or as recombinant mimotope fused to yellow fluorescent protein (mimotope-YFP) [42]. Analysis of binding kinetics revealed that the phage-mimotope is not ideal suited for robust assay development due to the large size of biologically active phages. In comparison to the mimotope-YFP, the synthetic mimotope shows a slightly better assay sensitivity but a lower affinity to the target antibody. As a consequence, this might be important for the improved sensitivity of competitive assays since a smaller amount of the analyte is required to compete with the synthetic mimotope for antibody binding. In contrast, Zhao et al. [43] coupled a synthetic mimotope directly to horseradish peroxidase (mimotope-HRP) and used it as tracer reagent in a direct, competitive assay format for AFB1 detection. The direct immunoassay applying mimotope-HRP is not only more sensitive than the traditional immunoassay set-up but also has the superiority of being faster and simpler with better performance than an indirect mimotope-based immunoassay. Nevertheless, all of the described assays applying epitope-mimicking molecules are established exclusively for single toxin detection. Moreover, the assays are time-consuming (assay times between 30 min and 3.5 h) and need a lot of hands-on-time by the operator in contrast to the automated multiplex biochip assay based on anti-idiotypic antibodies developed in this work.

Beside application of epitope-mimicking molecules as assay reagents, alternative recognition elements were also used for assay development to determine low molecular weight toxins by directly capturing the analyte of interest (Table S3). The use of viable cells was described by Li et al. [27]. The duplex assay is very straightforward, needs no further reagents but exhibits a four-times higher LOD (5.19 ng/mL STX) than the LOD reported here (1.2 ng/mL STX). However, the use of viable cells or natural receptor molecules offers a detection of all relevant congeners of a toxin group and often better reflects the in vivo toxicity of low molecular weight toxins compared to antibody-based methods. Molecularly imprinted polymers (MIPs) [26] and aptamers [25] have already been identified as stable compounds suitable for use in direct assays. Contrary to the enzyme-amplified immuno-based sensor developed here, which involves labeling of detection antibodies and multiple assay steps of reagent addition as well as intermediate washing steps, aptamer- and polymer-based sensors can be easily employed in label-free detection formats. Moreover, their inherent stability and low cost offer several advantages for biosensor application. Apart from that, MIPs often deal with significant lower affinities to the analyte as compared to antibodies. Therefore, LODs of MIP-based assays are often not sufficient to detect trace amounts of the analyte of interest [26]. Zejli et al. [25] overcome this limitation by developing a highly sensitive electrochemical aptasensor for AFB1 with a LOD of 1.6 pg/mL. However, the often high specificity of selected aptamers can be detrimental for on-site detection systems, which need to assess as many congeners as possible in a single run. This requirement is fulfilled by the here developed multiplex assay employing toxin group specific antibodies. Looking ahead, as more and more assays are being generated with alternative recognition elements or epitope-mimicking molecules, the possibility of replacing toxins in diagnostic procedures with presumably non-toxic reagents result in enhanced laboratory safety. This advantage could be even more significant when diagnostic test kits are used in the field by unskilled operators.

The here presented microfluidic fiveplex biochip assay in combination with the electrochemical detection platform is ideal suited for the on-site use since it is fully portable and can be deployed to any location required. The developed multiplex assay is quick (assay time: 13.4 min) and at the same time highly sensitive (LODs in the low ng/mL- to pg/mL-range). Moreover, processing of the biochip on the automated detection platform with minimal hands-on time for the end-user meets the demand for easy-to-use on-site systems to rapidly analyze suspicious samples. In contrast to our assay, recently published microfluidic biosensors for on-site toxin detection utilize traditional toxin-protein conjugates as capture or detection reagent (Table S3). For example, an ultrafast multiplex mycotoxin detection was achieved through oriented immobilization of toxin specific antibodies with protein G beads packed in microchannels (assay time: 1 min) [44]. However, application to sample matrices revealed that the fluorescence signal was highly susceptible to matrix interferences. In contrast, the multiplex assay developed here is capable to detect qualitatively multiple low molecular weight toxins from spiked human serum samples with only minimal sample preparation demonstrating the robustness of electrochemical detection systems [45]. Furthermore, previously published results demonstrated the applicability of singleplex electrochemical biosensors for analysis of low molecular weight toxins in urine samples representing another relevant body fluid. As an example of an robust optical system, Brickman et al. [46] reported a waveguide biosensor allowing a higher sensitivity compared to the here reported LOD for parallel detection of MC-LR (0.4 ng/mL) and cylindrospermopsin (0.7 ng/mL) on a portable and automated reader. Moreover, the short range of the evanescent wave enables it to discriminate between bound and unbound fluorescent labeled detection molecules, hence, eliminating the need for washing steps. Another novel system for point-of-need testing relies on a disc-based biosensor with recombinantly produced antibodies for simultaneous determination of MC-LR, STX and domoic acid within 30 min with LODs of 7.2 ng/mL, 20 ng/mL and 30 ng/mL, respectively [47]. Here, our assay achieves dramatically lower LODs for MC-LR (1.5 ng/mL) and STX (1.2 ng/mL) within a significant shorter assay time, thus demonstrating the exceptional ability of the here developed multiplex assay based on anti-idiotypic antibodies as epitope-mimicking molecules for developing a new generation of user-safe assays for low molecular weight toxins.

## 4. Conclusions

The proposed fiveplex biochip assay offers the possibility of an automated and rapid on-site detection of highly toxic low molecular weight toxins with LODs in the low ng/mL- to pg/mL-range as well as broad specificity for congeners of the corresponding toxin group. For example, it can be applied for determining qualitatively the presence or absence of the bioterrorism relevant toxins STX, MC-LR, RoA, HT-2 and AFB1 in human serum samples after a suspected deliberate release of a biological warfare agent. By relying solely on anti-idiotypic antibodies as epitope-mimicking molecules, no derivatization of low molecular weight toxins for immobilization or conjugation to a reporter enzyme is necessary. Notably, this work demonstrates the applicability of anti-idiotypic antibodies to develop user-safe competitive immunoassays with superior analytical performance, including lower LODs and IC_50_ as well as a much higher degree of multiplexing capability than previously reported assays based on other epitope-mimicking molecules or alternative recognition elements. Furthermore, the application of antibody-reporter enzyme conjugates as tracers shortens the overall assay time to 13.4 min which allow a more straightforward assay workflow. The biochip will be automatically processed on a portable detection platform facilitating minimal end-user interaction ideal for on-site testing. The toxin group specificity of the antibodies enables that as many congeners as possible can be detected in a single analysis. In combination with the existing electrochemical biochip assays for high molecular weight toxins (e.g., botulinum neurotoxins, ricin) as well as for bioterrorism relevant microorganisms (e.g., *Bacillus anthracis*, *Yersinia pestis*) [36], the pBDi platform is a valuable tool for rapid and straightforward on-site detection of various kinds of biothreat agents. Moreover, the detection system has the flexibility to combine these assays for different kinds of biological agents on one biochip without modifying the general workflow, thus, offering a new level of biothreat detection.

Further work is in progress to extend the use of this fiveplex biochip assay in different fields of application, e.g., for food and environmental analysis.

## 5. Materials and Methods

### 5.1. Materials

Reagents: methanol, Tween-20 (T-20), trehalose, magnesium chloride hexahydrate (MgCl_2_), sodium dihydrogen phosphate (NaH_2_PO_4_), disodium hydrogen phosphate (Na_2_HPO_4_), sodium chloride (NaCl), phosphate-buffered saline pH 7.4 (1× PBS; 10 mM Na_2_HPO_4_, 137 mM NaCl, 1.8 mM potassium dihydrogen chloride, 2.7 mM potassium chloride), thimerosal, 2-mercaptoethanol (2-ME), skimmed milk powder, sodium hydroxide (NaOH), sodium dodecyl sulfate (SDS), bovine serum albumin (BSA), *o*-nitrophenyl-β-D-galactopyranoside (ONPG), pAPG, bGAL, SA-bGAL, Amicon Ultra-50K MWCO centrifugal filter devices, anti-mouse IgG-bio and polyclonal rabbit anti-mouse IgG (anti-mouse IgG) were all purchased from Sigma-Aldrich GmbH (Taufkirchen, Germany). Mouse IgG were obtained from Dianova GmbH (Hamburg, Germany). Zeba Spin-7K MWCO desalting columns and sulfo-succinimidyl-4-(N-maleimidomethyl)cyclohexane-1-carboxylate (sulfo-SMCC) were from Thermo Fisher Scientific (Waltham, MA, USA). Water used in all experiments was purified with a Milli-Q purification system (Millipore, Billerica, MA, USA).

Toxin standards: cyanotoxins standards, including STX, dc-STX, dc-NEO, GTX-1/-4, dc-GTX-2/-3,C1/C2, MC-LR, MC-RR, MC-YR and nodularin-R were all purchased from LabMix24 GmbH (Hamminkeln, Germany). NEO, GTX-2/-3 and GTX-5 were all from CIFGA S.A. (Lugo, Spain). MC-LA, MC-LY, MC-LW and [DAsp^3^]MC-LR were all purchased from Enzo Life Sciences (Farmingdale, NY, USA). T-2 and its metabolites HT-2, T-2 triol and T-2 tetraol were all obtained from Romer Labs GmbH (Getzersdorf, Austria). Verrucarol, RoA, VerA as well as AFB1, AFG1, AFM1, AFB2 and AFG2 were all from Sigma-Aldrich GmbH (Taufkirchen, Germany). RoE was purchased from Biozol Diagnostica GmbH (Eching, Germany).

### 5.2. Methods

#### 5.2.1. Antibody Production

The utilized mAbs1 were as follows: anti-STX mAb 5F7 (STX/mAb1), anti-MC-LR mAb 4H6 (MC-LR/mAb1), anti-T-2 mAb 2A12 (T-2/mAb1), anti-RoA mAb 7F8 (RoA/mAb1) and anti-AFB1 mAb 2D1 (AFB1/mAb1) and prepared as previously described [39,48]. The corresponding mAb2s were used as follows: anti-5F7 mAb 1D8 (STX/mAb2), anti-4H6 mAb 6A11 (MC-LR/mAb2), anti-2A12 mAb 1D6 (T-2/mAb2), anti-7F8 mAb 5G11 (RoA/mAb2) and anti-2D1 mAb 1G10 (AFB1/mAb2) and generated as previously reported [48,49].

#### 5.2.2. Immobilization of Capture Antibodies on Gold Electrodes of Biochips

The biochips (9 mm × 10 mm) were manufactured on 8-inch silicon wafers at the Fraunhofer Institute for Silicon Technology (Itzehoe, Germany) exhibiting 16 IDA gold electrodes (diameter: 500 µm) as described [50]. Capture Abs were applied to the biochip via non-contact piezo-electronic spotter sciFLEXARRAYER S3 (Scienion AG, Berlin, Germany) as reported by Schulz et al. [35]. Spotting concentrations were 400 µg/mL STX/mAb2, 200 µg/mL MC-LR/mAb2, 100 µg/mL T-2/mAb1, 200 µg/mL RoA/mAb2, 400 µg/mL AFB1/mAb1 in 0.4 mg/mL BSA/1× PBS, 2.5 µg/mL anti-mouse IgG-bio in 0.2 mg/mL BSA/1× PBS, and 500 µg/mL mouse IgG in 0.4 mg/mL BSA/1× PBS. BSA served as a co-immobilization agent to stabilize the capture Abs on the gold electrode surface. After spotting, biochips were treated as previously described [35].

#### 5.2.3. Conjugation of Detection Antibodies to β-D-Galactosidase

Detection mAbs STX/mAb1, MC-LR/mAb1, T-2/mAb2, RoA/mAb1 and AFB1/mAb2 were covalently coupled to bGAL via an amine-to-sulfhydryl cross linkage. Antibody activation was carried out by adding a 20-fold molar excess of crosslinking reagent sulfo-SMCC (4.8 mg/mL in water) to 1 mg detection mAb (concentrated to 2 mg/mL in conjugation buffer (0.1 M sodium phosphate pH 7.2, 0.1 M NaCl). After incubation for 1 h at room temperature, excess of non-reacted sulfo-SMCC was removed using Zeba Spin-7K MWCO desalting columns equilibrated with conjugation buffer. For formation of stable thioether bonds between the maleimide-activated mAb (mAb-SMCC) and free sulfhydryl groups in bGAL, a two-fold molar excess of mAb-SMCC (2 mg/mL in conjugation buffer) was mixed with bGAL (3.7 mg/mL in 1× PBS) and incubated overnight at 4 °C. The resulting mAb-bGAL tracers STX/mAb1-bGAL, MC-LR/mAb1-bGAL, T-2/mAb2-bGAL, RoA/mAb1-bGAL and AFB1/mAb2-bGAL were finally mixed with 1% (*w*/*v*) BSA (stabilizer) as well as 0.01% (*v*/*v*) thimerosal (preservative) and stored at 4 °C. Conjugation was confirmed by microplate-based immunoassay. In brief, serial dilutions of mAb-bGAL conjugates were applied to microtiter plates pre-coated with anti-mouse IgG (5 µg/mL in 1× PBS). Detection was realized by adding colorimetric bGAL substrate ONPG (2 mg/mL in 1× PBS, 1 mM MgCl_2_, 10 Mm 2-ME).

#### 5.2.4. Instrumentation and Electrochemical Biochip Measurements

Spotted biochips inserted in a polycarbonate cartridge were processed and measured using the electrochemical detection platform pBDi (Bruker Optik GmbH, Leipzig, Germany). For the electrochemical measurement, a potential of +150 mV to the anodic fingers and −400 mV to the cathodic fingers of the biochip IDA gold electrodes was applied and current readout was achieved within a range of ±200 nA.

For multiplex detection of STX, MC-LR, T-2, RoA and AFB1, a direct competitive immunoassay was performed. Toxin sample was mixed with mAb-bGAL tracer cocktail (1:2500 STX/mAb1-bGAL, 1:3500 MC-LR/mAb1-bGAL, 1:750 T-2/mAb2-bGAL, 1:6000 RoA/mAb1-bGAL and 1:7000 AFB1/mAb2-bGAL) and SA-bGAL (0.22 U/mL), yielding a total volume of 800 µL. The mixture was immediately applied to the detection platform and automatically analyzed. The automatic program summarized in Table S2 has an overall assay time of 13.4 min and includes, amongst other steps, washing steps with assay buffer (1× PBS, 0.5% (*w*/*v*) BSA, 0.5% trehalose, 1 mM MgCl_2_, 0.025% (*v*/*v*) T-20), a competition step of free toxins in the sample with mAbs2 for limited binding sites of the corresponding mAbs1 as well as PC binding reaction and, finally, a signal generating step applying the bGAL substrate pAPG (1 mg/mL in assay buffer). Monitoring of the generated current by enzymatic substrate conversion and subsequent redox cycling was performed according to Elsholz et al. [50] in stopped flow mode (Figure S3). The absolute current slope of target electrode positions was normalized to the signal of the spotted PC and NC according to Equation (1):

(1) $$\mathrm{Normalized}\;\mathrm{signal}\;\left(\%\right)=100\times \left[\frac{\mathrm{Slope}\left(\mathrm{Target}\right)-\mathrm{Slope}\left(\mathrm{NC}\right)}{\mathrm{Slope}\left(\mathrm{PC}\right)-\mathrm{Slope}\left(\mathrm{NC}\right)}\right]$$

Normalized signals were used for further data analysis.

#### 5.2.5. Assay Specificity

Cross-reactivity of the developed assay was determined by incubating upon the fiveplex biochip toxin solutions with 100 ng/mL STX, T-2, MC-LR, RoA or AFB1, respectively, in the presence of mAb-bGAL tracer cocktail under competitive conditions.

Specificity of the applied mAb pairs to toxin group congeners was assessed by analyzing toxin solutions containing either 100 ng/mL of a PSP-toxin (STX, dc-STX, NEO, dc-NEO, GTX-1/-4, GTX-2/-3, dc-GTX-2/-3, GTX-5, C1/C2), 50 ng/mL of a MC (MC-LR, [DAsp^3^]MC-LR, MC-RR, MC-YR, MC-LA, MC-LY, MC-LW, nodularin-R), 100 ng/mL of a type A trichothecene (T-2, HT-2, T-2 triol, T-2 tetraol, verrucarol), 50 ng/mL of a type D trichothecene (RoA, RoE, VerA, SatH) or 50 ng/mL of an aflatoxin (AFB1, AFG1, AFM1, AFB2, AFG2) upon the fiveplex biochip.

Measurements were performed in assay buffer.

#### 5.2.6. Assay Sensitivity

Sensitivity of the fiveplex biochip assay was evaluated by testing toxin standard solutions prepared in assay buffer using various concentrations ranging from 0–600 ng/mL STX, 0–300 ng/mL MC-LR, AFB1, T-2 or HT-2, respectively, and 0–200 ng/mL RoA.

#### 5.2.7. Simultaneous Toxin Detection

For simultaneous toxin detection, toxin standard solutions (STX, MC-LR, T-2, RoA and AFB1) were prepared at four different concentration levels (IC_5_, LOD, IC_50_, IC_80_) in assay buffer and applied to the biochip.

#### 5.2.8. Analysis of Toxins Spiked in Human Blood Serum

Human serum from male AB clotted whole blood (Sigma-Aldrich GmbH, Taufkirchen, Germany) was mixed with STX (0, 6, 20, 200 ng/mL), MC-LR (0, 6, 20, 200 ng/mL), HT-2 (0, 4, 10, 100 ng/mL), RoA (0, 4, 10, 100 ng/mL) and AFB1 (0, 4, 10, 100 ng/mL) to prepare spiked serum samples at four different concentration levels (zero, low, medium, high). Serum samples were shaken at 400 rpm for 1 h at room temperature to address potential matrix effects. Afterwards, samples were diluted two-fold (concentration level low and mid) or ten-fold (concentration level high) in assay buffer and directly subjected to biochip analysis. Even if the original sample matrix can generally vary in factors such as salt concentrations, viscosity, protein content or pH appropriate dilution of sample matrix in assay buffer can adjust these parameters to optimal assay conditions for the established multiplex assay.

#### 5.2.9. Data Analysis

Dose-response curves were described by the four-parameter logistic regression model using GraphPad Prism 6 (GraphPad Software, La Jolla, CA, USA). Displayed error bars represent the SD of mean target specific signals (n) obtained from different biochips as indicated. One biochip was employed for the analysis of one sample. LOD, working range (IC_30_–IC_80_), and midpoint (IC_50_) were interpolated from the four-parameter logistic function. The LOD corresponds to the toxin concentration which gave a normalized signal equal to the normalized signal when no toxin is present (B0) minus three times of its SD obtained from *n* = 20. The working range was determined as the concentrations of the toxin reducing the B0 signal to 30–80%. The IC_50_ was calculated as the toxin concentration that lowered the normalized signal to 50% of B0. Percent inhibition was calculated using Equation (2):

(2) $$\%I=100-\left[\frac{\mathrm{Signal}\;\times \;100}{B0\;\mathrm{Signal}}\right]$$

For recovery calculation, %(B/B0)_Serum_-values were used for interpolation from dose-response curves obtained in assay buffer.

## Figures and Tables

**Table 1:** Specificity of the employed antibodies to toxin group congeners. Antibody specificity is classified according to the signal reduction efficiency (%I) of the fiveplex biochip assay after applying (i) 100 ng/mL of a PSP toxin, (ii) 50 ng/mL of a MC or nodularin-R, (iii) 100 ng/mL of a type A trichothecene, (iv) 50 ng/mL of a type D trichothecene and (v) 50 ng/mL of an aflatoxin, respectively (*n* = 4, i.e., two biochips with each two target electrode positions): +++ high (%I: 100–70%); ++ moderate (%I: <70–40%); + low (%I: <40–10%); − negligible (%I: <10%).

Toxins		Target Electrode Positions for:				
Group	Congener	STX	MC-LR	T-2	RoA	AFB1
PSP toxin	STX	+++	−	−	−	−
	NEO	−	−	−	−	−
	GTX-1/-4	−	−	−	−	−
	dc-STX	++	−	−	−	−
	GTX-2/-3	+++	−	−	−	−
	GTX-5	++	−	−	−	−
	dc-NEO	−	−	−	−	−
	dc-GTX-2/-3	+++	−	−	−	−
	C1/C2	−	−	−	−	−
MC	MC-LR	−	+++	−	−	−
	[DAsp^3^]MC-LR	−	+++	−	−	−
	MC-RR	−	+++	−	−	−
	MC-YR	−	+++	−	−	−
	MC-LA	−	+	−	−	−
	MC-LY	−	+	−	−	−
	MC-LW	−	+	−	−	−
Nodularin	Nodularin-R	−	+++	−	−	−
Type A trichothecene	T-2	−	−	+++	−	−
	HT-2	−	−	+++	−	−
	T-2 triol	−	−	−	−	−
	T-2 tetraol	−	−	−	−	−
	Verrucarol	−	−	−	−	−
Type D trichothecene	RoA	−	−	−	+++	−
	RoE	−	−	−	+++	−
	SatH	−	−	−	++	−
	VerA	−	−	−	+++	−
Aflatoxin	AFB1	−	−	−	−	+++
	AFM1	−	−	−	−	+++
	AFG1	−	−	−	−	+++
	AFB2	−	−	−	−	++
	AFG2	−	−	−	−	++

Abbreviation: NEO = neosaxitoxin, GTX-1/-4 = gonyautoxin 1 and 4, dc-STX = decarbamoylsaxitoxin, GTX-2/-3 = gonyautoxin 2 and 3, GTX-5 = gonyautoxin 5, dc-NEO = decarbamoylneosaxitoxin, dc-GTX-2/-3 = decarbamoylgonyautoxin 2 and 3, C1/C2 = N-sulfocarbamoyl-gonyautoxin 2 and 3, RoE = roridin E, SatH = satratoxin H, VerA = verrucarin A, AFG1 = aflatoxin G1, AFB2 = aflatoxin B2, AFG2 = aflatoxin G2.

**Table 2:** Sensitivity and reproducibility of the fiveplex biochip assay for detection of STX, MC-LR, T-2, HT-2 and RoA as well as AFB1. Inter-chip %CV was determined from ten independent experiments (*n* = 20, i.e., ten biochips with each two target electrode positions). B0 = zero standard; B_IC50_ = IC_50_ standard.

Toxin	Sensitivity (ng/mL)			Reproducibility (Inter-Chip %CV)	
	LOD	IC_50_	IC_30_–IC_80_	B0	B_IC50_
STX	1.2	5.9	1.8–56.4	9.9	10.4
MC-LR	1.5	5.5	2.8–31.1	9.2	10.2
T-2	0.4	1.4	0.5–17.4	10.1	9.2
HT-2	0.7	2.4	0.8–25.6		11.6
RoA	0.5	1.2	0.5–10.8	9.6	10.0
AFB1	0.6	1.7	0.6–10.5	11.2	8.2

**Table 3:** Number of correctly identified serum samples as well as recovery rates for the detection of various concentrations of STX, MC-LR, HT-2, RoA and AFB1 spiked in human serum samples with inter-chip SD, mean %(B/B0)_Serum_-values and frequency of correctly identified samples obtained from five independent experiments. In total, 20 biochips were used for the analysis of serum samples. Spiked serum samples were diluted to obtain an assay concentration within the working range.

Toxin	Spiking Concentration (ng/mL)	Assay Concentration (ng/mL)	%(B/B0)_Serum_ * (% ± SD)	Recovery Rate (% ± SD)	Correctly Identified (%)
STX	0	0	96.7 ± 13.9	-	100
	6	3	60.6 ± 6.5	113.7 ± 24.5	100
	20	10	41.7 ± 8.8	103.2 ± 16.11	100
	200	20	32.2 ± 3.6	112.6 ± 26.1	100
MC-LR	0	0	110.9 ± 19.8	-	100
	6	3	78.7 ± 6.5	78.8 ± 22.7	80
	20	10	46.9 ± 10.6	93.9 ± 24.9	100
	200	20	26.3 ± 4.8	98.2 ± 22.5	100
HT-2	0	0	117.3 ± 20.4	-	100
	4	2	67.5 ± 4.9	51.8 ± 17.6	100
	10	5	54.3 ± 10.6	60.6 ± 8.9	100
	100	10	26.3 ± 3.5	79.2 ± 24.3	100
RoA	0	0	101.4 ± 18.9	-	100
	4	2	38.9 ± 3.8	87.9 ± 6.9	100
	10	5	24.7 ± 2.2	100.7 ± 13.4	100
	100	10	16.7 ± 1.7	95.6 ± 18.2	100
AFB1	0	0	109.6 ± 17.1	-	100
	4	2	48.7 ± 6.3	101.1 ± 31.1	100
	10	5	25.6 ± 5.1	112.9 ± 24.6	100
	100	10	17.4 ± 2.9	114.8 ± 18.43	100

* For non-spiked samples (0 ng/mL), %(B0_Serum_/B0_Buffer_)-values are shown.

## Supplementary Materials

Following tables are available online at https://www.mdpi.com/2072-6651/11/12/696/s1, Figure S1: Selection of the capture mAb for the detection of (A) MC-LR and (B) RoA. Figure S2: Raw data and mean absolute slopes as well as mean normalized slopes used for determination of assay specificity. Figure S3: Amperogram of all 16 electrode positions for a zero standard measurement (B0). Table S1: Optimal concentration of mAb1/mAb2-pairs for simultaneous detection of STX, MC-LR, T-2, RoA and AFB1. Table S2: Main steps in the automated assay program for simultaneous toxin detection utilizing a direct, competitive immunoassay (total assay time: 13.4 min). Table S3: Comparison of the here reported fiveplex biochip assay with other recently published methods for the detection of low molecular weight toxins employing epitope-mimicking molecules or alternative recognition elements or traditional toxin-protein conjugates for assay development.
