# Supplementary material for: An Electrochemical Fiveplex Biochip Assay Based on Anti-Idiotypic Antibodies for Fast On-Site Detection of Bioterrorism Relevant Low Molecular Weight Toxins
Source: Toxins (Basel). 2019 Nov 28;11(12):696. doi: 10.3390/toxins11120696 (PMC6950599; doi:10.3390/toxins11120696)
Supplement: Supplementary file 1 [file toxins-11-00696-s001.pdf]

Supplementary Materials: An Electrochemical Fiveplex Biochip Assay Based on Anti-Idiotypic Antibodies for Fast On-Site Detection of Bioterrorism Relevant Low Molecular Weight Toxins

Katharina Schulz, Christopher Pöhlmann, Richard Dietrich, Erwin Märtlbauer and Thomas Elßner

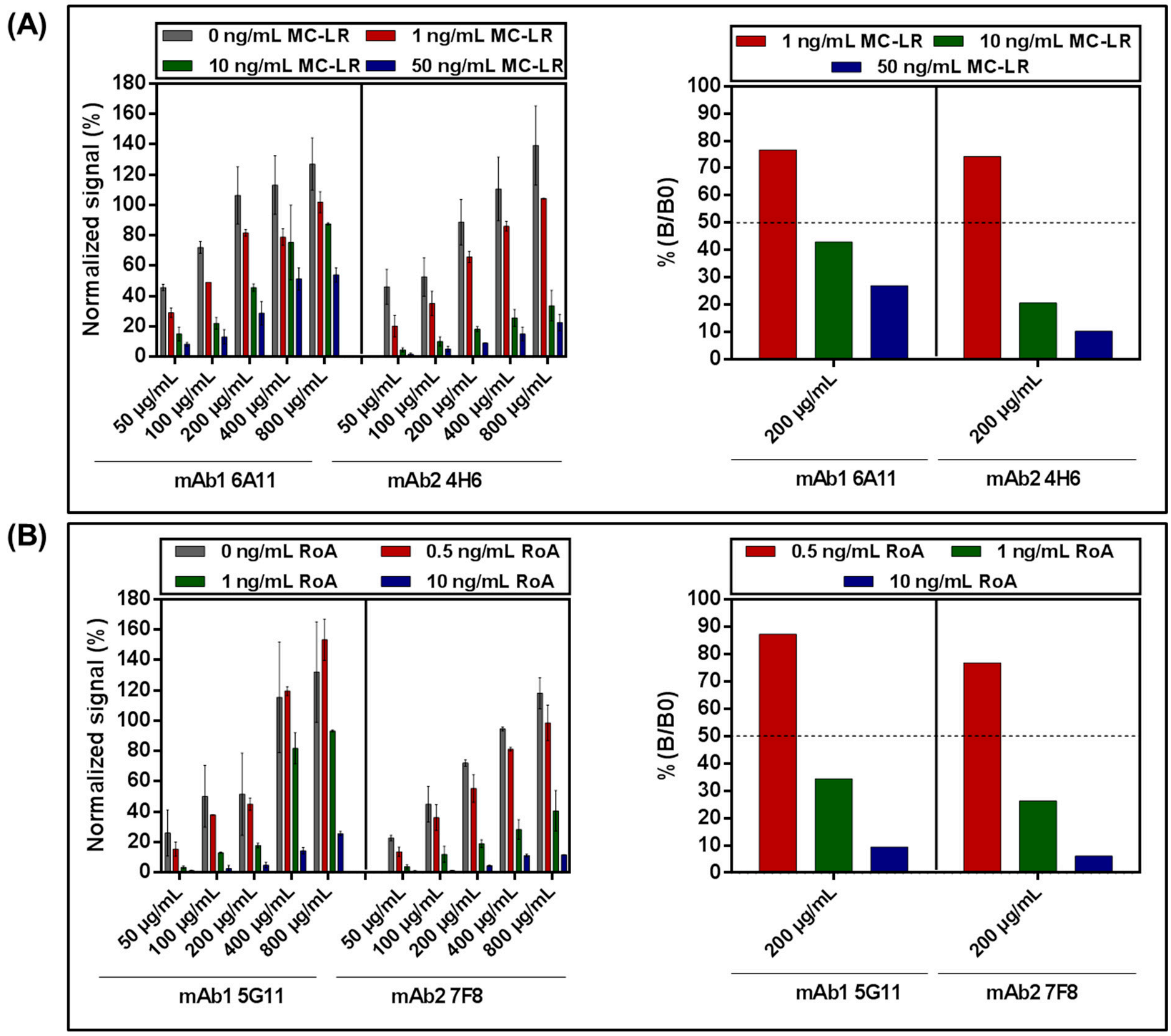

Figure S1. Selection of the capture mAb for the detection of (**A**) MC-LR and (**B**) RoA. Left: Influence of the capture mAb concentration on the assay signal (n = 4, i.e., two biochips with each two target electrode positions). Right: Influence of the mAb type used as capture on assay sensitivity. Experiments were performed in singleplex format applying varied toxin concentrations and a defined concentration of the respective detection mAb. Detection mAb concentrations were (**A**) 150 ng/mL and (**B**) 300 ng/mL (n = 4, i.e., two biochips with each two target electrode positions). The selection of the most suitable capture mAb was performed in singleplex experiments applying the indirect competitive biochip assay with electrical biochips spotted with varying concentrations of mAb1 or mAb2, different toxin dilutions and a defined concentration of the corresponding detection mAb conjugated to biotin as described previously for STX,T-2 and aflatoxins [1].

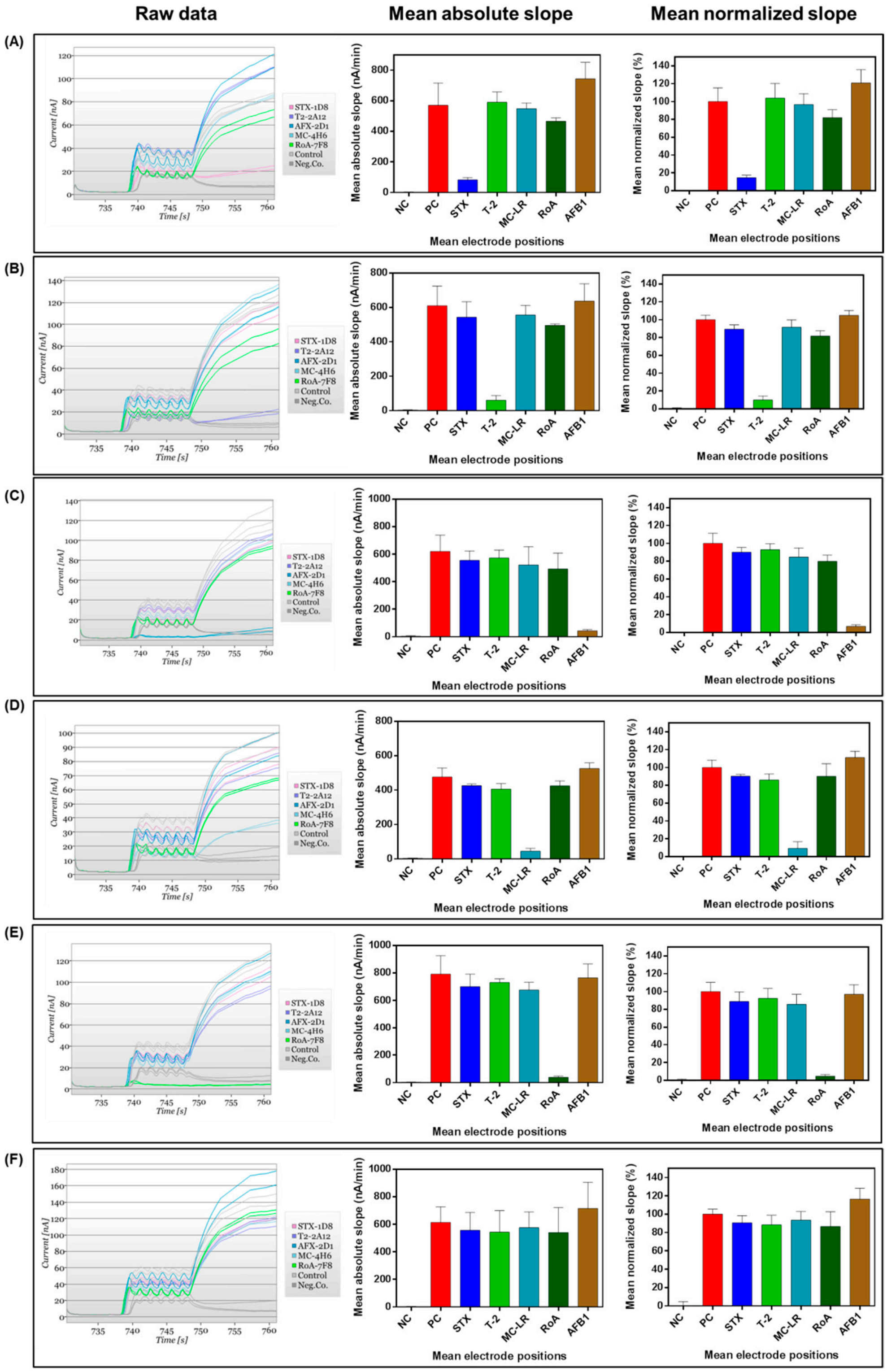

Figure S2. Raw data and mean absolute slopes as well as mean normalized slopes used for determination of assay specificity. Data were obtained from (**A**) 100 ng/mL STX, (**B**) 100 ng/mL T-2, (**C**) 100 ng/mL AFB1, (**D**) 100 ng/mL MC-LR, (**E**) 100 ng/mL RoA and (**F**) B0 (zero standard). Mean slope values were obtained from four independent measurements (n = 8, i.e., four biochips with each two target electrode positions). To calculate percent inhibition, mean normalized slope values were used.

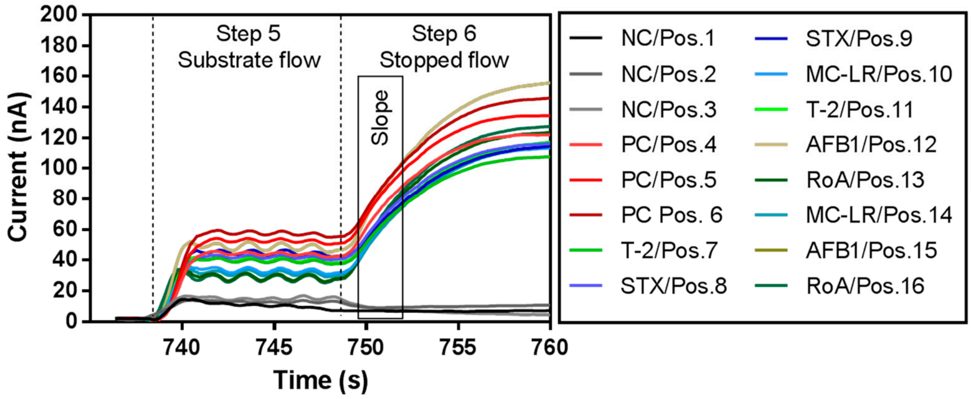

Figure S3. Amperogram of all 16 electrode positions for a zero standard measurement (B0). Following sample application and competition reaction as well as washing steps with assay buffer, the substrate is applied automatically to the processed biochip during the assay program in step 5. Then, the substrate flow is stopped (step 6) and electrochemical measurement is performed. After a delay of 1 sec, a 4 sec-measuring interval in the stopped flow mode is used to determine the absolute slope value for each electrode position by linear regression. For a better batch-to-batch comparison of different biochip production runs, the absolute slope value of each electrode position is normalized to the signals of the positive control (PC) and negative control (NC).

Table S1. Optimal concentration of mAb1/mAb2-pairs for simultaneous detection of STX, MC-LR, T-2, RoA and AFB1.

Toxin	Capture mAb		Detection mAb-bGAL Conjugate	
	**mAb Type**	**Concentration (µg/mL)**	**mAb Type**	**Dilution**
STX	mAb2 1D8	400	mAb1 5F7-bGAL	1:2500
MC-LR	mAb2 4H6	200	mAb1 6A11-bGAL	1:3500
T-2	mAb1 2A12	100	mAb2 1D6-bGAL	1:750
RoA	mAb2 7F8	200	mAb1 5G11-bGAL	1:6000
AFB1	mAb1 2D1	400	mAb2 1G10-bGAL	1:7000

Table S2. Main steps in the automated assay program for simultaneous toxin detection utilizing a direct, competitive immunoassay (total assay time: 13.4 min).

Step	Process	Duration (s)	Temperature (°C)
1	Equilibration with assay buffer	49	32
2	Sample + mAb-bGAL tracer cocktail flow	20	42
3	Competition reaction	594	42
4	Wash flow with assay buffer	74	42
5	Substrate flow	10	50
6	Stopped flow	15	50
7	Wash flow with assay buffer	41	32

Table S3. Comparison of the here reported fiveplex biochip assay with other recently published methods for the detection of low molecular weight toxins employing epitope-mimicking molecules or alternative recognition elements or traditional toxin-protein conjugates for assay development.

**Toxin**	**Assay Characteristics**					**Integration in A Detection Platform**		**Sample**	**Ref.**
	**Assay Principle**	**Detection**	**LOD**	**Multi-plex**	**Assay Time**	**Fully Automated**	**Portable**		
**Epitope-Mimicking Molecules**									
STX<br>MC-LR<br>T-2<br>RoA<br>AFB1	IDA gold electrode applying mAb1/mAb2-pairs as capture and detector	Electro-chemical	1.2 ng/mL<br>1.5 ng/mL<br>0.4 ng/mL<br>0.5 ng/mL<br>0.6 ng/mL	Yes	13.4 min	Yes	Yes	Serum	This study
FB1	Immunoassay with Ab2-Nb as capture and mAb1 with HRP-labeled secondary Ab as detector	Absorbance	0.15 ng/mL	No	1 h	No	No	Food, feed	[2]
AFB1	Magnetic-bead based assay with Ab1-Nb as capture and mimotope-HRP as tracer	Absorbance	0.13 ng/mL	No	35 min	No	No	Food	[3]
FB1	Microarray with immobilized synthetic mimotope and mAb1 as detector in combination with fluorophore-labeled secondary Abs	Fluores-cence	11.1 ng/mL	No	3.5 h	No	No	Maize, wheat	[4]
**Alternative Recognition Elements**									
STX<br>DA	Cell-based sensor using a combined IDA gold and potential electrode seeded with cardiomyocytes	Electro-chemical	5.19 ng/mL<br>7.16 ng/mL	Yes	30 min	No	Yes	Buffer	[5]

AFB1	Nanostructured AFB1-MIP membrane	Intrinsic Fluores-cence	14 ng/mL	No	1 h	No	No	Waste water	[6]
AFB1	SPCE array immobilizing PT3C/MB-tagged aptamer	Electro-chemical	1.6 pg/mL	No	45 min	No	No	Coffee	[7]
**Traditional Toxin-Protein Conjugates**									
AFB1<br>OA<br>DON	Thin-film photodiode array with protein G beads bound anti-toxin Abs and fluorophore coupled BSA-toxin conjugates	Fluores-cence	1 ng/mL<br>3 ng/mL<br>10 ng/mL	Yes	1 min	Yes	Yes	Corn	[8]
MCs<br>CYN	Planar waveguide biosensor with toxin-BSA conjugates and fluorophore coupled mAbs	Fluores-cence	0.4 ng/mL<br>0.7 ng/mL	Yes	10 min	Yes	Yes	Lake water	[9]
MC-LR<br>STX<br>DA	Disc-based biosensor with surface bound toxins and fluorophore labeled anti-toxin rAbs	Fluores-cence	7.2 ng/mL<br>20 ng/mL<br>30 ng/mL	Yes	30 min	Yes	Yes	Lake water	[10]

Abbreviation: LOD = limit of detection; Ref. = reference; STX = saxitoxin; MC = microcystin; T-2 = T-2 toxin; RoA = roridin A; AFB1 = aflatoxin B1; FB1 = fumonisin B1; DA = domoic acid; OA = okadaic acid; CYN = cylindrospermopsin; OTA = ochratoxin A; ZEN = zearalenon; DON = deoxynivalenol; IDA = interdigitated array; SPCE = screen printed carbon electrode; Ab = antibody; mAb = monoclonal antibody; mAb1 = monoclonal toxin specific antibody; mAb2 = monoclonal anti-idiotypic antibody; Ab2-Nb = anti-idiotypic nanobody; Ab1-Nb = toxin specific nanobody; rAb = recombinant antibody; HRP = horseradish peroxidase; BSA = bovine serum albumin; PT3C = polythiophene-3-carboxylic acid; MB = methylene blue; MIP = molecularly imprinted polymer.

References

1. Schulz, K.; Pöhlmann, C.; Dietrich, R.; Märtlbauer, E.; Elßner, T. Electrochemical Biochip Assays Based on Anti-idiotypic Antibodies for Rapid and Automated On-Site Detection of Low Molecular Weight Toxins. *Front. Chem.* **2019**, *7*, doi:10.3389/fchem.2019.00031.
2. Shu, M.; Xu, Y.; Wang, D.; Liu, X.; Li, Y.; He, Q.; Tu, Z.; Qiu, Y.; Ji, Y.; Wang, X. Anti-idiotypic nanobody: A strategy for development of sensitive and green immunoassay for Fumonisin B1. *Talanta* **2015**, *143*, 388–393, doi:10.1016/j.talanta.2015.05.010.
3. Zhao, F.; Tian, Y.; Shen, Q.; Liu, R.; Shi, R.; Wang, H.; Yang, Z. A novel nanobody and mimotope based immunoassay for rapid analysis of aflatoxin B1. *Talanta* **2019**, *195*, 55–61, doi:10.1016/j.talanta.2018.11.013.
4. Peltomaa, R.; Benito-Pena, E.; Barderas, R.; Sauer, U.; González Andrade, M.; Moreno-Bondi, M.C. Microarray-Based Immunoassay with Synthetic Mimotopes for the Detection of Fumonisin B1. *Anal. Chem.* **2017**, *89*, 6216–6223, doi:10.1021/acs.analchem.7b01178.
5. Li, H.; Wei, X.; Gu, C.; Su, K.; Wan, H.; Hu, N.; Wang, P. A Dual Functional Cardiomyocyte-based Hybrid-biosensor for the Detection of Diarrhetic Shellfish Poisoning and Paralytic Shellfish Poisoning Toxins. *Anal. Sci.* **2018**, *34*, 893–900, doi:10.2116/analsci.18P029.
6. Sergeyeva, T.; Yarynka, D.; Piletska, E.; Lynnik, R.; Zaporozhets, O.; Brovko, O.; Piletsky, S.; El'skaya, A. Fluorescent sensor systems based on nanostructured polymeric membranes for selective recognition of Aflatoxin B1. *Talanta* **2017**, *175*, 101–107, doi:10.1016/j.talanta.2017.07.030.
7. Zejli, H.; Goud, K.Y.; Marty, J.L. An electrochemical aptasensor based on polythiophene-3-carboxylic acid assisted methylene blue for aflatoxin B1 detection. *Sens. Biosensing Res.* **2019**, *25*, 100290, doi:10.1016/j.sbsr.2019.100290.
8. Soares, R.R.G.; Santos, D.R.; Pinto, I.F.; Azevedo, A.M.; Aires-Barros, M.R.; Chu, V.; Conde, J.P. Multiplexed microfluidic fluorescence immunoassay with photodiode array signal acquisition for sub-minute and point-of-need detection of mycotoxins. *Lab Chip* **2018**, *18*, 1569–1580, doi:10.1039/c8lc00259b.
9. Bickman, S.R.; Campbell, K.; Elliott, C.; Murphy, C.; O'Kennedy, R.; Papst, P.; Lochhead, M.J. An Innovative Portable Biosensor System for the Rapid Detection of Freshwater Cyanobacterial Algal Bloom Toxins. *Environ. Sci. Technol.* **2018**, *52*, 11691–11698, doi:10.1021/acs.est.8b02769.
10. Maguire, I.; Fitzgerald, J.; Heery, B.; Nwankire, C.; O'Kennedy, R.; Ducree, J.; Regan, F. Novel Microfluidic Analytical Sensing Platform for the Simultaneous Detection of Three Algal Toxins in Water. *ACS omega* **2018**, *3*, 6624–6634, doi:10.1021/acsomega.8b00240.
